# (Nitrato-κ*O*)oxido(5,10,15,20-tetra­phenyl­porphyrinato-κ^4^ *N*)molybdenum(V) benzene solvate

**DOI:** 10.1107/S1600536808030705

**Published:** 2008-09-27

**Authors:** Shawn M. Carter, Nan Xu, Masood A. Khan, Douglas R. Powell, George B. Richter-Addo

**Affiliations:** a Department of Chemistry and Biochemistry, University of Oklahoma, 620 Parrington Oval, Norman, OK 73019, USA

## Abstract

In the title compound, [Mo(C_44_H_28_N_4_)(NO_3_)O]·C_6_H_6_, the porphyrin ring is centrosymmetric. The Mo atom, oxide ion and nitrate ion are equally disordered over two sites, such that the Mo atom is displaced by 0.366 (1) Å towards the oxide ion from the 24-atom mean plane of the porphyrin, and also makes a long Mo—O bond to a nitrate O atom. A centrosymmetric benzene solvent mol­ecule is situated between adjacent porphyrin mol­ecules.

## Related literature

For the structure of (TPP)Mo(O)(ONO_2_) (TPP is the tetraphenylporphyrinate dianion) with CH_2_Cl_2_ as the solvate, see: Okubo *et al.* (1999 ▶). For the crystal structures of related molybdenum(V)-oxo porphyrin complexes, see: Harada *et al.* (2004 ▶); Kim *et al.* (1987 ▶); Hamstra *et al.* (1999 ▶); Fujihara *et al.* (2002 ▶); Ledon & Mentzen (1978 ▶); Liu *et al.* (2001 ▶); Imamura & Furusaki (1990 ▶); Cheng & Scheidt (1996 ▶).
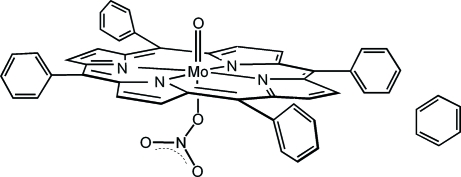


## Experimental

### 

#### Crystal data

[Mo(C_44_H_28_N_4_)(NO_3_)O]·C_6_H_6_
*M* *_r_* = 864.76
Triclinic, 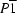

*a* = 8.6846 (14) Å
*b* = 11.2895 (18) Å
*c* = 11.7180 (18) Å
α = 61.617 (5)°
β = 79.283 (6)°
γ = 76.354 (6)°
*V* = 978.6 (3) Å^3^
*Z* = 1
Mo *K*α radiation
μ = 0.39 mm^−1^
*T* = 120 (2) K
0.16 × 0.08 × 0.02 mm

#### Data collection

Bruker SMART APEX CCD diffractometer
Absorption correction: multi-scan (*SADABS*; Sheldrick, 2007 ▶) *T*_min_ = 0.938, *T*_max_ = 0.994
10263 measured reflections
3836 independent reflections
3395 reflections with *I* > 2σ(*I*)
*R*_int_ = 0.023

#### Refinement

*R*[*F* ^2^ > 2σ(*F* ^2^)] = 0.049
*wR*(*F* ^2^) = 0.108
*S* = 1.16
3836 reflections
298 parameters
3 restraints
H-atom parameters constrained
Δρ_max_ = 0.43 e Å^−3^
Δρ_min_ = −0.47 e Å^−3^

### 

Data collection: *SMART* (Bruker, 1998 ▶); cell refinement: *SAINT* (Bruker, 1998 ▶); data reduction: *SAINT*; program(s) used to solve structure: *SHELXTL* (Sheldrick, 2008 ▶); program(s) used to refine structure: *SHELXTL*; molecular graphics: *SHELXTL*; software used to prepare material for publication: *SHELXTL*.

## Supplementary Material

Crystal structure: contains datablocks I, global. DOI: 10.1107/S1600536808030705/hb2799sup1.cif

Structure factors: contains datablocks I. DOI: 10.1107/S1600536808030705/hb2799Isup2.hkl

Additional supplementary materials:  crystallographic information; 3D view; checkCIF report

## Figures and Tables

**Table 1:** Selected bond lengths (Å)

Mo1—O1	1.678 (9)
Mo1—N1^i^	2.039 (2)
Mo1—N2^i^	2.044 (2)
Mo1—N2	2.139 (2)
Mo1—N1	2.159 (2)
Mo1—O2	2.227 (9)

Symmetry code: (i) 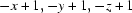.

**Fig. 1.**
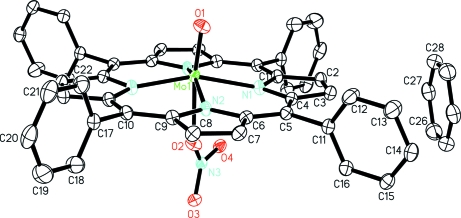
The molecular structure of (I) with displacement ellipsoids drawn at the 30% probability level (H atoms omitted for clarity). The unlabelled atoms of the complex and the benzene molecule are generated by the symmetry operations (1-x, 1-y, 1-z) and (-x, -y, 1-z), respectively.

## supplementary crystallographic information

### Comment

In this paper, we report the structure of the title compound, (I), a six-coordinate nitrate molybdenum(V)-oxo tetraphenylporphyrin with benzene as solvate, and the new approach to synthesize the compound using oxochloromolybdenum tetraphenylporphyrin as the precursor. The structure of the related compound with CH_2_Cl_2_ as solvate has been reported previously (Okubo *et al.* 1999).

The molecular structure of (I) is shown in Fig. 1. Both the metal complex and the benzene molecule were found to sit on a crystallographic center of symmetry. The porphyrin core of the compound has a slight wave shape. The Mo atom is displaced by 0.366 (1) Å from the 24-atom mean porphyrin plane toward the oxo ion. The nitrate ligand binds to the molybdenum atom through one of its oxygen atoms. Selected bond lengths are given in Table 1. The Mo(V)═O distance of 1.678 (9) Å in (I) is in the range of those [1.658 (2)–1.722 (6) Å] reported previously for other molybdenum(V)-oxo porphyrin complexes (Harada *et al.*, 2004; Kim *et al.*, 1987; Hamstra *et al.*, 1999; Fujihara *et al.*, 2002; Ledon & Mentzen, 1978; Liu *et al.*, 2001; Imamura & Furusaki, 1990; Cheng & Scheidt, 1996). The O═Mo—O linkage in (I) is essentially linear with a bond angle of 174.4 (16)°. A benzene molecule is situated between two adjacent porphyrin molecules with 1:1 benzene/porphyrin stoichiometry.

### Experimental

To a toluene solution (20 ml) of (TPP)Mo(O)Cl (0.015 g, 0.020 mmol) (Ledon & Mentzen, 1978) under nitrogen was added LiAlH_4_ (0.0016 g, 0.42 mmol) (Aldrich Chemical Company, used as received). Then, nitric oxide (98%; Matheson Gas, purified by passing through KOH pellets and a cold trap (dry ice/acetone) to remove higher nitrogen oxides) was bubbled through the mixture for 15 min. The mixture was stirred for an additional 30 min and filtered. A dark green solid was obtained after removal of solvent under vacuum. A suitable dark green prism-shaped crystal of (I) was grown by slow evaporation of a benzene solution of the product at room temperature under nitrogen.

### Refinement

The H atoms were positioned geometrically (C—H = 0.95 Å) and refined as riding with U_iso_(H) = 1.2U_eq_(C). The metal, the oxo ion and the nitrate group are disordered by 50% across the center of symmetry.

### Crystal data

**Table:**

[Mo(C_44_H_28_N_4_)(NO_3_)O]·C_6_H_6_	*Z* = 1
*M**_r_* = 864.76	*F*(000) = 443
Triclinic, *P*1	*D*_x_ = 1.467 Mg m^−^^3^
Hall symbol: -P 1	Mo *K*α radiation, λ = 0.71073 Å
*a* = 8.6846 (14) Å	Cell parameters from 7414 reflections
*b* = 11.2895 (18) Å	θ = 3.2–26.3°
*c* = 11.7180 (18) Å	µ = 0.39 mm^−^^1^
α = 61.617 (5)°	*T* = 120 K
β = 79.283 (6)°	Prism, green
γ = 76.354 (6)°	0.16 × 0.08 × 0.02 mm
*V* = 978.6 (3) Å^3^	

### Data collection

**Table:**

Bruker SMART APEX CCD diffractometer	3836 independent reflections
Radiation source: fine-focus sealed tube	3395 reflections with *I* > 2σ(*I*)
graphite	*R*_int_ = 0.023
ω scans	θ_max_ = 26.0°, θ_min_ = 2.0°
Absorption correction: multi-scan (SADABS; Sheldrick, 2007)	*h* = −10→10
*T*_min_ = 0.938, *T*_max_ = 0.994	*k* = −13→13
10263 measured reflections	*l* = −14→14

### Refinement

**Table:**

Refinement on *F*^2^	Primary atom site location: structure-invariant direct methods
Least-squares matrix: full	Secondary atom site location: difference Fourier map
*R*[*F*^2^ > 2σ(*F*^2^)] = 0.049	Hydrogen site location: inferred from neighbouring sites
*wR*(*F*^2^) = 0.108	H-atom parameters constrained
*S* = 1.16	*w* = 1/[σ^2^(*F*_o_^2^) + (0.032*P*)^2^ + *P*] where *P* = (*F*_o_^2^ + 2*F*_c_^2^)/3
3836 reflections	(Δ/σ)_max_ < 0.001
298 parameters	Δρ_max_ = 0.43 e Å^−^^3^
3 restraints	Δρ_min_ = −0.47 e Å^−^^3^

### Special details

**Table:**

Geometry. All e.s.d.'s (except the e.s.d. in the dihedral angle between two l.s. planes) are estimated using the full covariance matrix. The cell e.s.d.'s are taken into account individually in the estimation of e.s.d.'s in distances, angles and torsion angles; correlations between e.s.d.'s in cell parameters are only used when they are defined by crystal symmetry. An approximate (isotropic) treatment of cell e.s.d.'s is used for estimating e.s.d.'s involving l.s. planes.
Refinement. Refinement of *F*^2^ against ALL reflections. The weighted *R*-factor *wR* and goodness of fit *S* are based on *F*^2^, conventional *R*-factors *R* are based on *F*, with *F* set to zero for negative *F*^2^. The threshold expression of *F*^2^ > σ(*F*^2^) is used only for calculating *R*-factors(gt) *etc*. and is not relevant to the choice of reflections for refinement. *R*-factors based on *F*^2^ are statistically about twice as large as those based on *F*, and *R*- factors based on ALL data will be even larger.

### Fractional atomic coordinates and isotropic or equivalent isotropic displacement parameters (Å^2^)

**Table:**

	*x*	*y*	*z*	*U*_iso_*/*U*_eq_	Occ. (<1)
Mo1	0.47549 (6)	0.53402 (4)	0.48115 (5)	0.02365 (15)	0.50
O1	0.354 (3)	0.668 (2)	0.380 (2)	0.031 (3)	0.50
O2	0.652 (3)	0.356 (2)	0.598 (2)	0.035 (3)	0.50
N3	0.6402 (5)	0.2554 (5)	0.7138 (5)	0.0285 (10)	0.50
O4	0.5111 (4)	0.2409 (4)	0.7791 (4)	0.0340 (9)	0.50
O3	0.7618 (5)	0.1698 (4)	0.7537 (4)	0.0369 (9)	0.50
N1	0.3480 (3)	0.3743 (2)	0.5196 (2)	0.0283 (5)	
N2	0.6227 (2)	0.4736 (2)	0.3433 (2)	0.0275 (5)	
C1	0.2198 (3)	0.3414 (3)	0.6109 (3)	0.0289 (6)	
C2	0.1423 (3)	0.2553 (3)	0.5895 (3)	0.0335 (6)	
H2	0.0503	0.2181	0.6382	0.040*	
C3	0.2241 (3)	0.2368 (3)	0.4875 (3)	0.0323 (6)	
H3	0.1994	0.1845	0.4516	0.039*	
C4	0.3545 (3)	0.3100 (3)	0.4433 (2)	0.0272 (6)	
C5	0.4674 (3)	0.3184 (3)	0.3386 (2)	0.0275 (6)	
C6	0.5898 (3)	0.3952 (3)	0.2919 (2)	0.0275 (6)	
C7	0.7039 (3)	0.4052 (3)	0.1842 (3)	0.0310 (6)	
H7	0.7085	0.3621	0.1307	0.037*	
C8	0.8043 (3)	0.4869 (3)	0.1715 (3)	0.0314 (6)	
H8	0.8924	0.5105	0.1083	0.038*	
C9	0.7534 (3)	0.5311 (3)	0.2710 (3)	0.0294 (6)	
C10	0.8261 (3)	0.6181 (3)	0.2904 (3)	0.0288 (6)	
C11	0.4563 (3)	0.2361 (3)	0.2711 (3)	0.0287 (6)	
C12	0.3794 (4)	0.2953 (3)	0.1587 (3)	0.0498 (9)	
H12	0.3345	0.3897	0.1219	0.060*	
C13	0.3678 (5)	0.2168 (3)	0.0993 (3)	0.0553 (10)	
H13	0.3158	0.2583	0.0214	0.066*	
C14	0.4304 (4)	0.0806 (3)	0.1518 (3)	0.0380 (7)	
H14	0.4194	0.0271	0.1121	0.046*	
C15	0.5086 (3)	0.0221 (3)	0.2614 (3)	0.0354 (6)	
H15	0.5536	−0.0722	0.2976	0.042*	
C16	0.5226 (3)	0.1001 (3)	0.3206 (3)	0.0326 (6)	
H16	0.5789	0.0588	0.3962	0.039*	
C17	0.9593 (3)	0.6766 (3)	0.1930 (3)	0.0300 (6)	
C18	1.1139 (4)	0.6125 (3)	0.2087 (3)	0.0480 (8)	
H18	1.1393	0.5289	0.2842	0.058*	
C19	1.2343 (4)	0.6692 (4)	0.1146 (4)	0.0599 (10)	
H19	1.3417	0.6248	0.1263	0.072*	
C20	1.1979 (4)	0.7891 (4)	0.0050 (3)	0.0495 (9)	
H20	1.2803	0.8280	−0.0590	0.059*	
C21	1.0445 (4)	0.8521 (3)	−0.0122 (3)	0.0480 (8)	
H21	1.0194	0.9340	−0.0892	0.058*	
C22	0.9241 (4)	0.7975 (3)	0.0823 (3)	0.0412 (7)	
H22	0.8172	0.8433	0.0707	0.049*	
C26	0.0430 (5)	−0.0977 (4)	0.6200 (4)	0.0581 (10)	
H26	0.0726	−0.1658	0.7037	0.070*	
C27	−0.0779 (4)	0.0081 (4)	0.6088 (4)	0.0515 (9)	
H27	−0.1319	0.0135	0.6850	0.062*	
C28	−0.1220 (4)	0.1060 (4)	0.4897 (4)	0.0566 (9)	
H28	−0.2066	0.1794	0.4823	0.068*	

### Atomic displacement parameters (Å^2^)

**Table:**

	*U*^11^	*U*^22^	*U*^33^	*U*^12^	*U*^13^	*U*^23^
Mo1	0.0272 (3)	0.0225 (3)	0.0250 (3)	−0.0065 (2)	0.0001 (2)	−0.0135 (3)
O1	0.043 (4)	0.030 (6)	0.011 (5)	−0.001 (4)	0.002 (3)	−0.006 (5)
O2	0.035 (4)	0.038 (6)	0.026 (8)	−0.004 (4)	−0.008 (5)	−0.008 (5)
N3	0.024 (2)	0.033 (3)	0.039 (3)	−0.005 (2)	0.002 (2)	−0.026 (3)
O4	0.035 (2)	0.028 (2)	0.037 (2)	−0.0094 (16)	0.0041 (17)	−0.0134 (18)
O3	0.036 (2)	0.029 (2)	0.044 (2)	−0.0025 (17)	−0.0056 (18)	−0.0156 (19)
N1	0.0291 (12)	0.0361 (13)	0.0240 (11)	−0.0124 (10)	0.0033 (9)	−0.0160 (10)
N2	0.0271 (12)	0.0343 (13)	0.0255 (11)	−0.0119 (9)	0.0022 (9)	−0.0156 (10)
C1	0.0284 (14)	0.0314 (14)	0.0267 (14)	−0.0088 (11)	0.0002 (11)	−0.0123 (12)
C2	0.0318 (15)	0.0360 (16)	0.0354 (16)	−0.0125 (12)	0.0014 (12)	−0.0167 (13)
C3	0.0351 (15)	0.0309 (15)	0.0348 (15)	−0.0095 (12)	−0.0004 (12)	−0.0172 (13)
C4	0.0297 (14)	0.0276 (14)	0.0252 (13)	−0.0069 (11)	−0.0018 (11)	−0.0120 (11)
C5	0.0311 (14)	0.0268 (14)	0.0251 (13)	−0.0055 (11)	−0.0026 (11)	−0.0117 (11)
C6	0.0306 (14)	0.0264 (14)	0.0237 (13)	−0.0040 (11)	−0.0020 (11)	−0.0102 (11)
C7	0.0389 (16)	0.0281 (14)	0.0264 (14)	−0.0062 (12)	0.0014 (12)	−0.0140 (12)
C8	0.0344 (15)	0.0308 (15)	0.0267 (14)	−0.0086 (12)	0.0042 (11)	−0.0122 (12)
C9	0.0301 (14)	0.0334 (15)	0.0252 (14)	−0.0080 (11)	0.0008 (11)	−0.0135 (12)
C10	0.0268 (14)	0.0320 (14)	0.0267 (14)	−0.0079 (11)	0.0017 (11)	−0.0125 (12)
C11	0.0310 (14)	0.0313 (14)	0.0266 (14)	−0.0109 (11)	0.0038 (11)	−0.0149 (12)
C12	0.075 (2)	0.0312 (16)	0.0470 (19)	0.0034 (16)	−0.0252 (17)	−0.0198 (15)
C13	0.083 (3)	0.0436 (19)	0.047 (2)	0.0021 (18)	−0.0304 (19)	−0.0239 (17)
C14	0.0465 (18)	0.0402 (17)	0.0360 (16)	−0.0147 (14)	0.0011 (13)	−0.0224 (14)
C15	0.0397 (16)	0.0299 (15)	0.0363 (16)	−0.0106 (12)	0.0029 (13)	−0.0148 (13)
C16	0.0373 (16)	0.0329 (15)	0.0275 (14)	−0.0085 (12)	−0.0033 (12)	−0.0123 (12)
C17	0.0328 (15)	0.0342 (15)	0.0290 (14)	−0.0126 (12)	0.0038 (11)	−0.0183 (12)
C18	0.0372 (18)	0.0411 (18)	0.049 (2)	−0.0083 (14)	0.0055 (14)	−0.0095 (15)
C19	0.0358 (19)	0.058 (2)	0.072 (3)	−0.0119 (16)	0.0178 (17)	−0.025 (2)
C20	0.061 (2)	0.048 (2)	0.048 (2)	−0.0306 (17)	0.0262 (17)	−0.0293 (17)
C21	0.069 (2)	0.0421 (18)	0.0316 (17)	−0.0245 (17)	0.0027 (15)	−0.0112 (14)
C22	0.0448 (18)	0.0402 (17)	0.0358 (17)	−0.0139 (14)	−0.0026 (14)	−0.0122 (14)
C26	0.064 (2)	0.046 (2)	0.056 (2)	−0.0140 (18)	−0.0220 (19)	−0.0079 (17)
C27	0.0438 (19)	0.062 (2)	0.057 (2)	−0.0220 (17)	−0.0022 (16)	−0.0294 (19)
C28	0.047 (2)	0.046 (2)	0.082 (3)	0.0000 (16)	−0.0212 (19)	−0.031 (2)

### Geometric parameters (Å, °)

**Table:**

Mo1—O1	1.678 (9)	C10—C17	1.493 (4)
Mo1—N1^i^	2.039 (2)	C11—C16	1.373 (4)
Mo1—N2^i^	2.044 (2)	C11—C12	1.380 (4)
Mo1—N2	2.139 (2)	C12—C13	1.391 (4)
Mo1—N1	2.159 (2)	C12—H12	0.9500
Mo1—O2	2.227 (9)	C13—C14	1.367 (4)
O2—N3	1.30 (2)	C13—H13	0.9500
N3—O4	1.232 (6)	C14—C15	1.361 (4)
N3—O3	1.241 (6)	C14—H14	0.9500
N1—C1	1.377 (3)	C15—C16	1.391 (4)
N1—C4	1.382 (3)	C15—H15	0.9500
N1—Mo1^i^	2.039 (2)	C16—H16	0.9500
N2—C9	1.377 (3)	C17—C18	1.366 (4)
N2—C6	1.385 (3)	C17—C22	1.381 (4)
N2—Mo1^i^	2.044 (2)	C18—C19	1.391 (4)
C1—C10^i^	1.398 (4)	C18—H18	0.9500
C1—C2	1.436 (4)	C19—C20	1.370 (5)
C2—C3	1.352 (4)	C19—H19	0.9500
C2—H2	0.9500	C20—C21	1.356 (5)
C3—C4	1.432 (4)	C20—H20	0.9500
C3—H3	0.9500	C21—C22	1.387 (4)
C4—C5	1.400 (4)	C21—H21	0.9500
C5—C6	1.395 (4)	C22—H22	0.9500
C5—C11	1.507 (3)	C26—C27	1.363 (5)
C6—C7	1.428 (4)	C26—C28^ii^	1.374 (5)
C7—C8	1.354 (4)	C26—H26	0.9500
C7—H7	0.9500	C27—C28	1.360 (5)
C8—C9	1.436 (4)	C27—H27	0.9500
C8—H8	0.9500	C28—C26^ii^	1.374 (5)
C9—C10	1.404 (4)	C28—H28	0.9500
C10—C1^i^	1.398 (4)		
			
O1—Mo1—N1^i^	101.6 (11)	C9—C8—H8	126.2
O1—Mo1—N2^i^	100.8 (9)	N2—C9—C10	126.3 (2)
N1^i^—Mo1—N2^i^	90.76 (8)	N2—C9—C8	108.4 (2)
O1—Mo1—N2	99.3 (8)	C10—C9—C8	125.3 (2)
N1^i^—Mo1—N2	88.93 (8)	C1^i^—C10—C9	125.7 (2)
N2^i^—Mo1—N2	159.56 (3)	C1^i^—C10—C17	117.6 (2)
O1—Mo1—N1	98.5 (11)	C9—C10—C17	116.7 (2)
N1^i^—Mo1—N1	159.74 (3)	C16—C11—C12	118.7 (3)
N2^i^—Mo1—N1	88.23 (8)	C16—C11—C5	120.4 (2)
N2—Mo1—N1	85.07 (8)	C12—C11—C5	120.9 (2)
O1—Mo1—O2	174.4 (16)	C11—C12—C13	120.0 (3)
N1^i^—Mo1—O2	77.5 (8)	C11—C12—H12	120.0
N2^i^—Mo1—O2	84.8 (7)	C13—C12—H12	120.0
N2—Mo1—O2	75.2 (7)	C14—C13—C12	120.6 (3)
N1—Mo1—O2	82.2 (8)	C14—C13—H13	119.7
N3—O2—Mo1	132.5 (17)	C12—C13—H13	119.7
O4—N3—O3	121.0 (5)	C15—C14—C13	119.6 (3)
O4—N3—O2	121.1 (11)	C15—C14—H14	120.2
O3—N3—O2	117.8 (10)	C13—C14—H14	120.2
C1—N1—C4	107.7 (2)	C14—C15—C16	120.1 (3)
C1—N1—Mo1^i^	126.60 (17)	C14—C15—H15	119.9
C4—N1—Mo1^i^	124.54 (17)	C16—C15—H15	119.9
C1—N1—Mo1	123.63 (17)	C11—C16—C15	120.8 (3)
C4—N1—Mo1	127.31 (17)	C11—C16—H16	119.6
C9—N2—C6	107.7 (2)	C15—C16—H16	119.6
C9—N2—Mo1^i^	126.73 (17)	C18—C17—C22	119.2 (3)
C6—N2—Mo1^i^	124.14 (17)	C18—C17—C10	122.1 (3)
C9—N2—Mo1	123.49 (17)	C22—C17—C10	118.7 (3)
C6—N2—Mo1	127.58 (17)	C17—C18—C19	120.3 (3)
N1—C1—C10^i^	126.1 (2)	C17—C18—H18	119.9
N1—C1—C2	108.4 (2)	C19—C18—H18	119.9
C10^i^—C1—C2	125.4 (2)	C20—C19—C18	120.0 (3)
C3—C2—C1	107.7 (2)	C20—C19—H19	120.0
C3—C2—H2	126.2	C18—C19—H19	120.0
C1—C2—H2	126.2	C21—C20—C19	120.0 (3)
C2—C3—C4	107.9 (2)	C21—C20—H20	120.0
C2—C3—H3	126.0	C19—C20—H20	120.0
C4—C3—H3	126.0	C20—C21—C22	120.3 (3)
N1—C4—C5	125.5 (2)	C20—C21—H21	119.9
N1—C4—C3	108.3 (2)	C22—C21—H21	119.9
C5—C4—C3	126.2 (2)	C17—C22—C21	120.2 (3)
C6—C5—C4	126.0 (2)	C17—C22—H22	119.9
C6—C5—C11	117.1 (2)	C21—C22—H22	119.9
C4—C5—C11	116.9 (2)	C27—C26—C28^ii^	120.0 (3)
N2—C6—C5	125.8 (2)	C27—C26—H26	120.0
N2—C6—C7	108.2 (2)	C28^ii^—C26—H26	120.0
C5—C6—C7	126.0 (2)	C28—C27—C26	120.6 (4)
C8—C7—C6	108.1 (2)	C28—C27—H27	119.7
C8—C7—H7	125.9	C26—C27—H27	119.7
C6—C7—H7	125.9	C27—C28—C26^ii^	119.3 (3)
C7—C8—C9	107.5 (2)	C27—C28—H28	120.3
C7—C8—H8	126.2	C26^ii^—C28—H28	120.3
			
N1^i^—Mo1—O2—N3	126 (3)	C3—C4—C5—C6	176.7 (3)
N2^i^—Mo1—O2—N3	34 (2)	N1—C4—C5—C11	178.0 (2)
N2—Mo1—O2—N3	−142 (3)	C3—C4—C5—C11	−3.8 (4)
N1—Mo1—O2—N3	−55 (2)	C9—N2—C6—C5	179.1 (2)
Mo1—O2—N3—O4	4(3)	Mo1^i^—N2—C6—C5	11.8 (4)
Mo1—O2—N3—O3	179.8 (15)	Mo1—N2—C6—C5	−13.2 (4)
O1—Mo1—N1—C1	−82.6 (9)	C9—N2—C6—C7	−0.2 (3)
N1^i^—Mo1—N1—C1	105.4 (2)	Mo1^i^—N2—C6—C7	−167.48 (17)
N2^i^—Mo1—N1—C1	18.0 (2)	Mo1—N2—C6—C7	167.58 (17)
N2—Mo1—N1—C1	178.7 (2)	C4—C5—C6—N2	1.7 (4)
O2—Mo1—N1—C1	103.0 (7)	C11—C5—C6—N2	−177.8 (2)
O1—Mo1—N1—C4	82.4 (9)	C4—C5—C6—C7	−179.2 (3)
N1^i^—Mo1—N1—C4	−89.6 (2)	C11—C5—C6—C7	1.3 (4)
N2^i^—Mo1—N1—C4	−177.0 (2)	N2—C6—C7—C8	0.5 (3)
N2—Mo1—N1—C4	−16.4 (2)	C5—C6—C7—C8	−178.7 (3)
O2—Mo1—N1—C4	−92.0 (7)	C6—C7—C8—C9	−0.7 (3)
O1—Mo1—N1—Mo1^i^	172.0 (9)	C6—N2—C9—C10	179.9 (3)
N1^i^—Mo1—N1—Mo1^i^	0.0	Mo1^i^—N2—C9—C10	−13.2 (4)
N2^i^—Mo1—N1—Mo1^i^	−87.39 (11)	Mo1—N2—C9—C10	11.6 (4)
N2—Mo1—N1—Mo1^i^	73.28 (10)	C6—N2—C9—C8	−0.2 (3)
O2—Mo1—N1—Mo1^i^	−2.4 (7)	Mo1^i^—N2—C9—C8	166.65 (18)
O1—Mo1—N2—C9	84.7 (11)	Mo1—N2—C9—C8	−168.61 (17)
N1^i^—Mo1—N2—C9	−16.9 (2)	C7—C8—C9—N2	0.6 (3)
N2^i^—Mo1—N2—C9	−106.2 (2)	C7—C8—C9—C10	−179.6 (3)
N1—Mo1—N2—C9	−177.5 (2)	N2—C9—C10—C1^i^	1.8 (4)
O2—Mo1—N2—C9	−94.2 (8)	C8—C9—C10—C1^i^	−178.0 (3)
O1—Mo1—N2—C6	−81.3 (11)	N2—C9—C10—C17	−175.5 (2)
N1^i^—Mo1—N2—C6	177.2 (2)	C8—C9—C10—C17	4.8 (4)
N2^i^—Mo1—N2—C6	87.9 (2)	C6—C5—C11—C16	96.5 (3)
N1—Mo1—N2—C6	16.6 (2)	C4—C5—C11—C16	−83.0 (3)
O2—Mo1—N2—C6	99.8 (8)	C6—C5—C11—C12	−83.6 (3)
O1—Mo1—N2—Mo1^i^	−169.1 (11)	C4—C5—C11—C12	96.9 (3)
N1^i^—Mo1—N2—Mo1^i^	89.31 (11)	C16—C11—C12—C13	1.2 (5)
N2^i^—Mo1—N2—Mo1^i^	−0.003 (2)	C5—C11—C12—C13	−178.7 (3)
N1—Mo1—N2—Mo1^i^	−71.32 (10)	C11—C12—C13—C14	0.7 (6)
O2—Mo1—N2—Mo1^i^	11.9 (8)	C12—C13—C14—C15	−1.8 (5)
C4—N1—C1—C10^i^	177.8 (3)	C13—C14—C15—C16	0.9 (5)
Mo1^i^—N1—C1—C10^i^	9.8 (4)	C12—C11—C16—C15	−2.1 (4)
Mo1—N1—C1—C10^i^	−14.7 (4)	C5—C11—C16—C15	177.9 (3)
C4—N1—C1—C2	−0.9 (3)	C14—C15—C16—C11	1.0 (4)
Mo1^i^—N1—C1—C2	−168.85 (18)	C1^i^—C10—C17—C18	92.1 (3)
Mo1—N1—C1—C2	166.57 (17)	C9—C10—C17—C18	−90.4 (3)
N1—C1—C2—C3	0.4 (3)	C1^i^—C10—C17—C22	−89.6 (3)
C10^i^—C1—C2—C3	−178.3 (3)	C9—C10—C17—C22	87.8 (3)
C1—C2—C3—C4	0.3 (3)	C22—C17—C18—C19	0.6 (5)
C1—N1—C4—C5	179.5 (2)	C10—C17—C18—C19	178.8 (3)
Mo1^i^—N1—C4—C5	−12.2 (4)	C17—C18—C19—C20	−0.7 (6)
Mo1—N1—C4—C5	12.7 (4)	C18—C19—C20—C21	−0.4 (6)
C1—N1—C4—C3	1.1 (3)	C19—C20—C21—C22	1.5 (5)
Mo1^i^—N1—C4—C3	169.33 (17)	C18—C17—C22—C21	0.5 (4)
Mo1—N1—C4—C3	−165.80 (17)	C10—C17—C22—C21	−177.8 (3)
C2—C3—C4—N1	−0.8 (3)	C20—C21—C22—C17	−1.6 (5)
C2—C3—C4—C5	−179.3 (3)	C28^ii^—C26—C27—C28	0.3 (6)
N1—C4—C5—C6	−1.5 (4)	C26—C27—C28—C26^ii^	−0.3 (6)

Symmetry codes: (i) −*x*+1, −*y*+1, −*z*+1; (ii) −*x*, −*y*, −*z*+1.
